# mRNA COVID-19 Vaccines—Facts and Hypotheses on Fragmentation and Encapsulation

**DOI:** 10.3390/vaccines11010040

**Published:** 2022-12-24

**Authors:** Jacques Demongeot, Cécile Fougère

**Affiliations:** AGEIS & Telecom4Health, Faculty of Medicine, University Grenoble Alpes, 38700 La Tronche, France

**Keywords:** microRNA, SARS-CoV-2, vaccine, mRNA, liposome, circular RNA

## Abstract

Background: The adventure of the mRNA vaccine began thirty years ago in the context of influenza. This consisted in encapsulating the mRNA coding for a viral protein in a lipid particle. We show how the mRNA encoding S protein has been modified for that purpose in the context of the anti-SARS-CoV-2 vaccination. Results: by using data coming from genetic and epidemiologic databases, we show the theoretical possibility of fragmentation of this mRNA into small RNA sequences capable of inhibiting important bio-syntheses such as the production of beta-globin. Discussion: we discuss two aspects related to mRNA vaccine: (i) the plausibility of mRNA fragmentation, and (ii) the role of liposomal nanoparticles (LNPs) used in the vaccine and their impact on mRNA biodistribution. Conclusion: we insist on the need to develop lipid nanoparticles allowing personalized administration of vaccines and avoiding adverse effects due to mRNA fragmentation and inefficient biodistribution. Hence, we recommend (i) adapting the mRNA of vaccines to the least mutated virus proteins and (ii) personalizing its administration to the categories of chronic patients at risk most likely to suffer from adverse effects.

## 1. Introduction

The chemistry of nucleic acids has experienced a great development since the deciphering of the human genome in 2003. This very important scientific advance has enabled the identification of new biological targets, responsible for numerous pathologies. Different therapeutic approaches can thus be developed now to control the specific expression of the genes responsible for some of these diseases, and mRNA has shown therapeutic potential in a wide range of applications, including viral vaccines, protein replacement therapies, cancer immunotherapies, as well as genome reprogramming and editing [1,2,3,4,5,6,7,8,9,10,11,12,13]. Over the past ten years, these RNA and DNA-based therapies have been developed, but most have not yet got marketing authorization and are still in phase I/II of clinical trial. At the same time, a great deal of research is looking into the manufacture of vaccines based on messenger RNA (mRNA) and DNA. However, the instability of oligonucleotides in biological media remains an obstacle to their use [14].

The first mRNA vaccine was developed in 1993 by Martinon et al., who described in [15] how they obtained this mRNA vaccine encoding an influenza virus protein, encapsulated in a nanoparticle liposome, in order to induce, in mice, the production of anti-influenza cytotoxic T lymphocytes (CTL).

A wide variety of chemical molecules was subsequently developed to deliver mRNA to cells, including lipids, lipid-like materials, polymers and protein derivatives [3,4,5,6,7,8,9,10,11,12,13]. Lipid nanoparticles have been clinically studied for the delivery of small molecules, drug-like siRNA [16] and mRNA [17,18,19], including two mRNAs, which have obtained conditional marketing authorization as vaccines against the coronavirus disease COVID-19, mRNA1273 [19] and BNT162b [20]. These vaccines use lipid nanoparticles to deliver mRNA encoding the viral spike protein. Many other lipid nanoparticles, as well as new mRNA formulas, have been developed and are undergoing clinical evaluation for the prevention and therapy of viral infections, cancer and genetic diseases [3,4,5,6,7,8,9,10,11,12,13].

In this article, we aim to explain the functioning of mRNA vaccines and to provide food for thought, in order to improve them to make them safer, taking into account the reactivity of viral RNA fragments (having possibly a microRNA-like inhibitory effect) and the bioavailability of their vectors.

## 2. Background

### 2.1. The Different RNA Strategies Considered in Therapy

**Figure 1:**
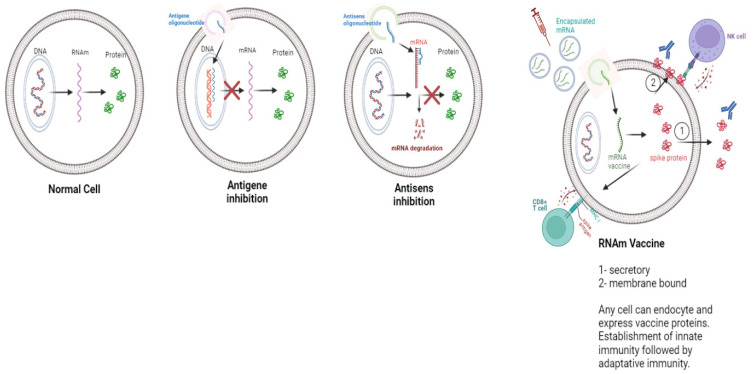
Different RNA or DNA based therapy strategies.

Different therapeutic approaches have been developed to control the specific expression of genes from oligonucleotides or oligonucleotide analogues. When they are directed against a messenger RNA (mRNA), the oligonucleotides inhibit the translation, referred to as “antisense” strategy (Figure 1). Antisense oligonucleotides were the first to be used [20,21]. The targets of oligonucleotides and their analogues can also be specific sequences of the DNA double helix. They then bind to it, forming a local triple helix, and inhibit the transcription, this is the “antigen” strategy. These strategies can be therapeutic (e.g., for the selective inhibition of oncogenes in tumor cells) or preventive (e.g., for the vaccination against infectious agents).

The principle of mRNA vaccines is based on the synthesis, by the host cells, of an antigenic protein, which will then be expressed on the surface of these cells [22].

### 2.2. Modifications in the mRNA Encoding the Spike Protein

DNAs and RNAs are rapidly degraded by enzymes (such as nucleases) in a biological environment [14]. In addition, oligonucleotides (RNA or DNA) have a poly-anionic structure, which results in a low cell penetration capacity. Over the past four decades, many oligonucleotide analogues have been synthesized to circumvent this difficulty. Most of the analogues (oligonucleotide mimics) studied have a structure close to natural oligonucleotides and, in general, chemical modifications on bases (A-T-G-C-U), sugars or phosphodiester skeleton, improve the biological stability of RNAs [23,24,25,26,27,28].

The anti-SARS-CoV-2 vaccines developed by the Pfizer/BioNTech^®^ (Pfizer, New York, USA and BioNTech, Mainz, Germany) and Moderna^®^ (Moderna, Cambridge, Massachusetts, USA) laboratories are based on the mRNA strategy encapsulated in a Nanoparticle Liposome (LNP). They relied on the synthesis, by our cells, of the viral protein Spike, in order to generate an immune response involving the cellular mechanisms of B and T lymphocytes. This protein is, in fact, the one that allows the virus to enter the cell via the ACE-2 [29,30] and NRP1 receptors [31]. In addition, this protein has been shown to cause many complications due to SARS-CoV-2 infection, by intervening in metabolic pathway of the renin-angiotensin system [32] and making this disease multi-systemic with at least possible complications, once in the cell. While viral spike protein mRNA vaccine has proven to be highly effective, its efficacy against emerging variants has decreased.

### 2.3. Specific Modification of the mRNA Sequence of the Pfizer/BioNtech Vaccine^®^

**Figure 2:**
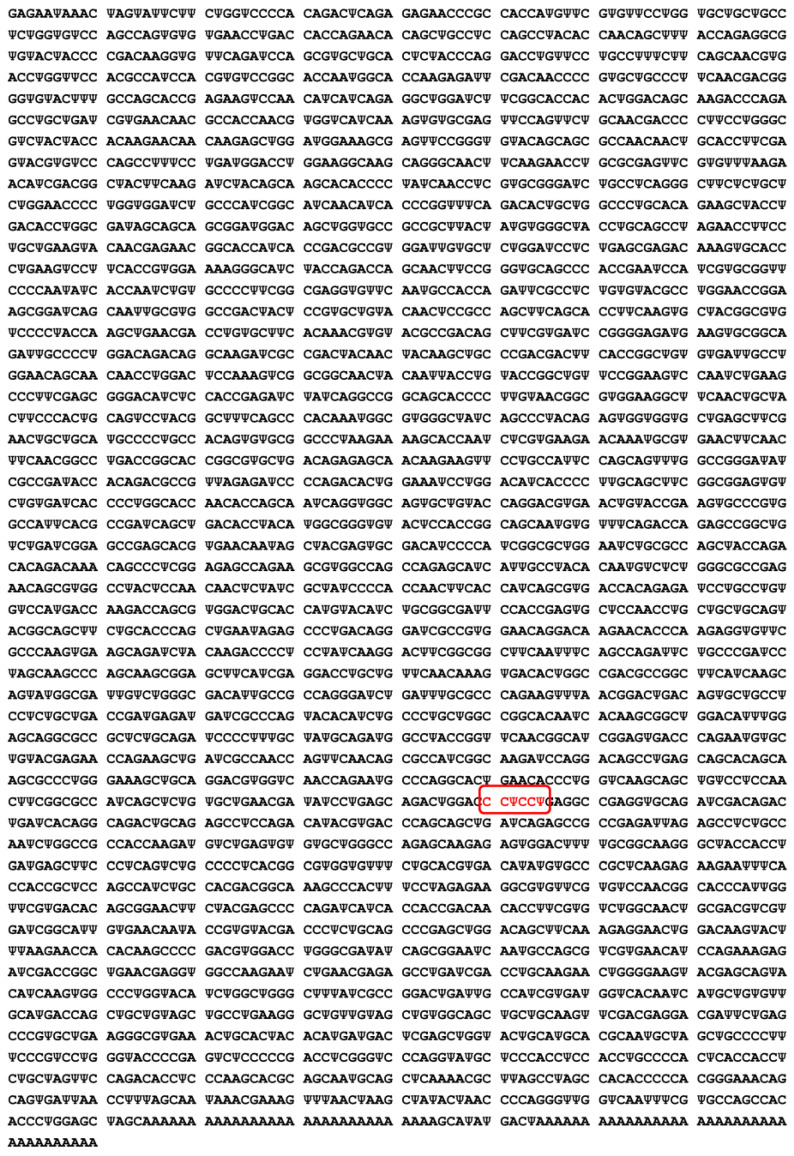
Sequence of the COVID-19 mRNA vaccine tozinameran (BNT162b2) from Pfizer/BioNTech^®^, where Ψ indicates N1-methyl-3′-pseudouridine. This sequence has 4250 nucleotides: Adenine A, Cytosine C, Guanine G and Pseudo-uridine Ψ. An amino-acid modification of the Spike protein mRNA is indicated in red.

The WHO posted the mRNA sequence of the Pfizer/BioNTech^®^ vaccine online in September 2020, which makes it possible to better understand the modifications made and why they were made [33] (Figure 2). We will then explain what these modifications are and what they cause, in particular in the bioavailability of the mRNA at the level of the target cells and in the existence of adverse effects observed within the target population.

### 2.4. Substitution of Uridine by N1-Methyl-3′-pseudouridine

**Figure 3:**
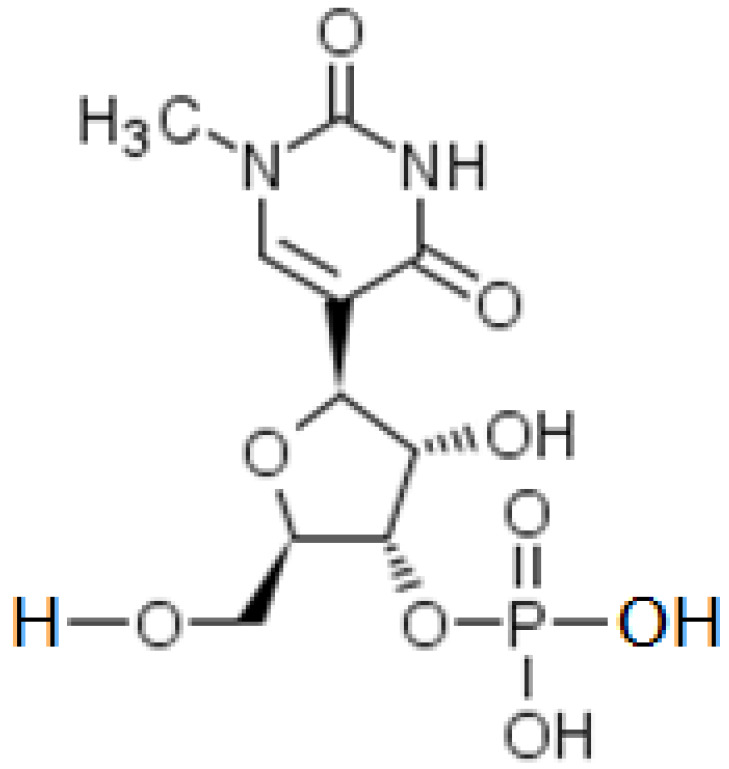
Semi-Structural formula of N1-methyl-3′-pseudo-uridine.

Studies report that exogenous free RNAs (coming from outside the cell) can provoke an innate immune response: indeed, the presence of foreign mRNAs in the cytoplasm and endosomes, as well as RNA-dependent kinases, named PKR, induce activation of the interferon signaling pathway. This action causes the production of pro-inflammatory cytokines, by activating the transcription factor NFκB [34]. Intracellular immune activation generates a reduced mRNA translation, due to a feedback between the innate immune system and the initiation of mRNA translation, which decreases the potency of therapeutic mRNA [34]. If not modified, this will result in a reduction in the number of proteins produced and a weakening of the immune response. This natural cellular defense mechanism aims to block the protein expression of a foreign mRNA gene. In order to overcome this problem, the Pfizer/BioNtech^®^ and Moderna^®^ laboratories have consequently chosen to substitute N1-methyl-3′-pseudouridine for the Uridine of the natural mRNA, in the mRNA sequence of the vaccine [33] (Figure 3).

The substitution of Uridine with N1-methyl-3′-pseudouridine decreases the inflammatory response. N1-methyl-3′-pseudouridine is the methylated derivative of pseudo-uridine. In vertebrates, it is known to significantly decrease, compared to Uridine, the innate immune response (inflammation). In addition, this modification leads to a more efficient translation of mRNA into a protein [34].

### 2.5. Modification of Codons and Substitution of 2 Amino Acids

Other modifications were made to the mRNA in order to make the translation of the protein more efficient and that it has the correct spatial conformation. We are going to detail these modifications made to the anti-SARS-CoV-2 vaccine mRNA and the consequences they can have. Several changes were thus made between the sequence present in the virus and the sequence present in the vaccine.

In Table 1, Lysine (K) and Valine (V) have been replaced by Proline (P). This modification was made so that the free Spike protein has the same spatial conformation as the protein Spike anchored in the membrane of the virus. If this had not been done, the antibodies generated could not have been effective. The interest of Prolines in the spatial conformation of free viral proteins has been known since 2017 [35]. Proline can appear in two different conformations CIS and TRANS [36] and stabilization of an intramolecular hydrogen bond makes it possible to control their distribution in a biological medium, ensuring the dominance of the TRANS conformation in 80% of cases [37].

As we can see in Table 1, some codons have been substituted by others, which code for the same amino acid. This additional modification increases the number of Cytosine (C) and Guanine (G) bases in the mRNA. The GC-rich genes were expressed several-fold to over a 100-fold more efficiently than their GC-poor counterparts [38].

### 2.6. mRNA and Poly(A) Tail Size

Polyadenylation, which consists of the addition of a Poly(A) tail, plays several essential roles during the different stages of the life of a messenger RNA [39]. It intervenes in particular in the protection against degradation, in the nucleo-cytoplasmic transport (passage of mRNA from the nucleus to the cytoplasm) and in the recruitment of the ribosome, to allow its translation into protein. Polyadenylation is also involved in excision of the last exon and plays a role in mRNA stability. Indeed, the Poly(A) tail modulates mRNA degradation in the cytosol. Whether for the mRNA vaccine from Pfizer/BioNtech^®^ laboratories or that of Moderna^®^, the two mRNA sequences end in a Poly(A) tail. The purpose of this addition is to increase the stability of the mRNA in biological medium and also to allow the recruitment of the ribosome, in order to initiate an efficient translation.

After translation, the mRNA can be reused several times, but when this happens, it also loses part of the Adenines of its Poly(A) tail, as enzymatic degradation begins there, which only ensures a transient protection against this degradation [40]. When this tail is too degraded, the mRNA is no longer functional and is destroyed. The poly(A) tail stabilizes mRNA and boosts protein translation, and the length of the poly(A) tail is proportional to translation efficiency. It is a critical factor in determining the longevity of mRNA molecules. [41] Mammalian cell mRNA molecules contain Poly(A) tails that are approximately 250 nucleotides in length, which gradually decrease in direction 3′ to 5′, during their life in the cytoplasm. Poly(A) tails of around 100 nucleotides have been shown to be ideal for mRNA-based therapies, since tail size modulates the 3′-exonucleolytic degradation of mRNA [42].

Deadenylation does not permanently inactivate the mRNA, because it can eventually be polyadenylated again. However, more often than not, when a certain number of “A” residues remaining on the mRNA is reached (on the order of 30 to 60 in mammals), the mRNA engages in rapid and irreversible degradation. For example, a long-tailed Poly(A) mRNA (such as hemoglobin mRNA) will be minimally degraded and therefore highly translated, allowing sustained production of the corresponding protein.

Conversely, histone mRNAs, which do not have a Poly(A) tail at all, are very rapidly degraded, allowing rapid modulation of histone expression across cell cycles, as there is no lingering mRNA translation inertia [39]. In the case of mRNA vaccines, the Poly(A) tail contains 120 Adenines. After transcription of the modified vaccine mRNA into protein, it is not completely degraded (due to the size of its Poly(A) tail), which allows it to be translated several times.

## 3. Micro-RNAs and Their Role

**Figure 4:**
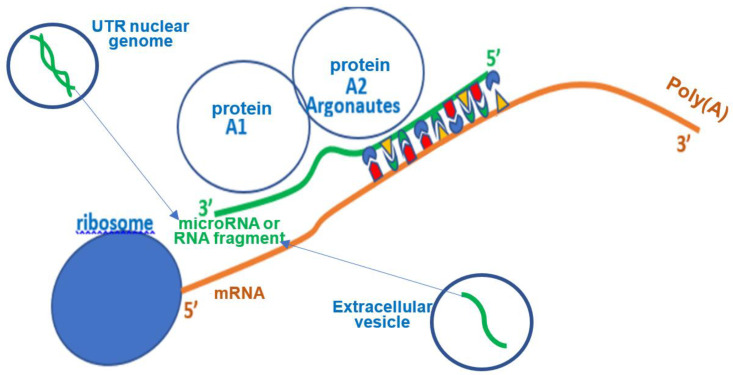
Synthetic representation of translation inhibition by small RNA.

A micro-RNA (miR) comes from a UTR (UnTranslated Region) nuclear DNA sequence, cleaved and fixed by the Dicer protein and exported into the cytoplasm in form of an oligonucleotide with a very large majority of 22 bases. It can then hybridize a mRNA being read in the ribosome, thus stopping the protein elongation (Figure 4). In animal cells, a miR does not hybridize completely with its target mRNA, but it is an effective inhibitor of ribosomal protein elongation, from 8 pairings at the 3′ end of this mRNA, before the tail Poly(A) (Figure 4). The pairing zone at the 5′ end of the miR is called the hybridization seed.

## 4. Results

### 4.1. Viral and Host Genomes

The viral genome is a source both, on a medical level, of techniques for detecting the presence of viruses in patients and monitoring their virulence and the effectiveness of the therapy, and, on a scientific level, of methods for studying the coevolution of viral genome with the genome of their host. In the first case, the COVID-19 pandemic is a good example of the massive use of detection techniques and, in the second, there are methods for calculating the proximity of the viral genome with the host or vector genome, for example the average match or concordance (percentage of common nucleotides) along the 5′-UTR of the viral genome, between this viral genome and all the miRs originating from a host or from a vector of the virus [43].

**Figure 5:**
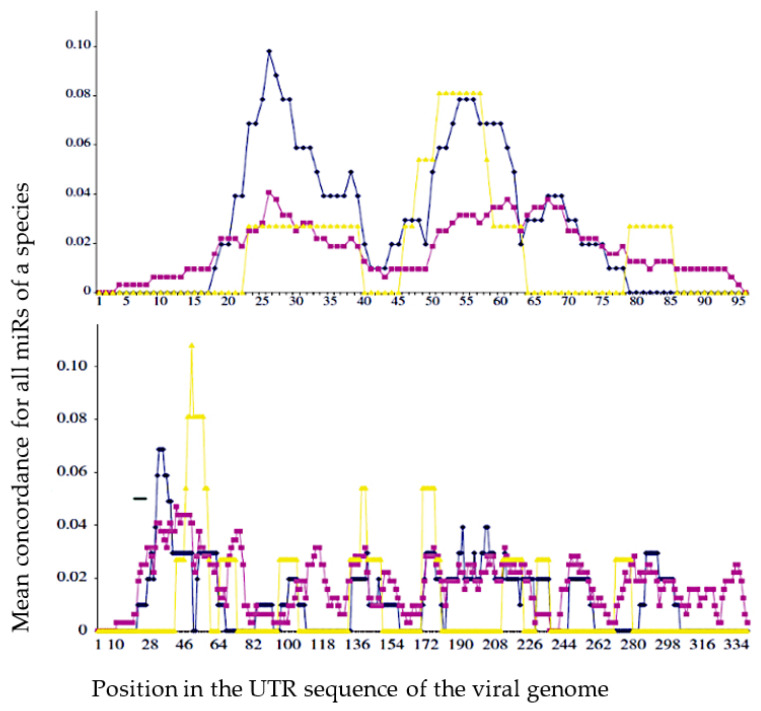
Mean concordance (in %) between the sequences of the miRs of the 5′-UTR part (UnTranslated Region) of the host genome (blue for G. gallus and purple for H. sapiens), vector genome (yellow for A. gambiae) and a sequence at the top, of West Nile virus (WNV) UTR genome and at the bottom, of hepatitis C virus (HCV) UTR genome (from [43]).

After calculating the average match for the hosts and vector in Figure 5, we notice a better agreement for the hosts whose coevolution with the virus has been the closest: we observe, for example, a better fit with Gallus gallus than with Homo sapiens for the West Nile virus (West Nile Virus) and the reverse for the hepatitis C virus (HCV). The existence of similarities between a viral genome and micro-RNAs from its host genome proved by a better match between sequences coming from their own infectious ecosystem than from others can be analyzed with not supervised classification techniques [44]. This point is important because it indicates that during their co-evolution viruses and hosts genomes can share common small nucleic sequences coming from nuclease and reverse transcriptase activity in host cells [45].

Cytoplasmic nucleases (e.g., RNases) are enzymes capable of cleaving the phosphodiester bonds of viral RNA and the viral genome fragments thus obtained can then form complexes with mRNAs and/or proteins in the host cell, preventing ribosomal translation of proteins just as miRs do [46]. Then, when RNA viruses replicate their capsid proteins in host cells and duplicate their genome, they leave behind RNA fragments, which can, if they bind to Argonaut proteins (which facilitate hybridization to mRNA and then its hydrolysis), behave as miRs in the host genome [47,48,49,50,51,52]. When the targeted proteins are vital, pathogenicity may thus be greater than that due to viral replication.

**Figure 6:**
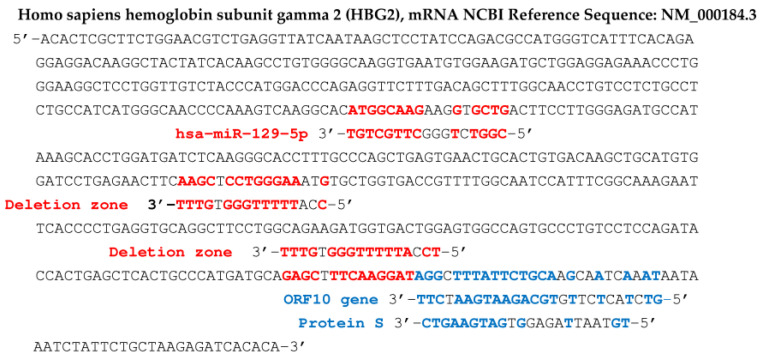
Human fetal hemoglobin gamma 2 subunit (HBG2) gene. Sequences in blue (resp. red) come from ORF10 and protein S genes and from the deletion zone of SARS-CoV-2 (resp. from hsa miR 129-5p). Probability of length 8 hybridization by chance in red (resp. 9, 11 in blue) for 577 nucleotides is equal to 0.035 (resp. 0.017, 0.0003), T-G and G-T hybridizations counting for ½.

**Figure 7:**
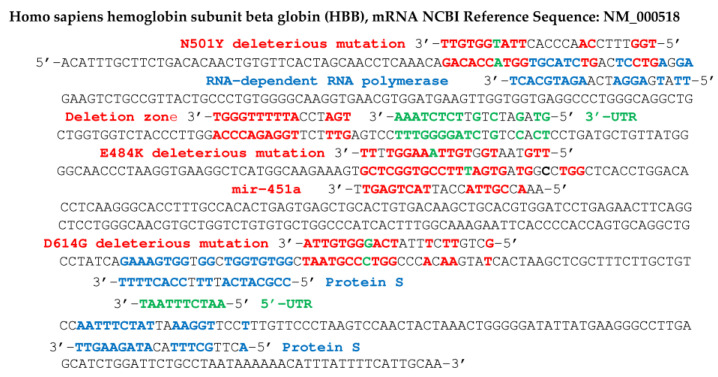
Human hemoglobin beta-subunit (HBB) gene potentially targeted by SARS-CoV-2 gene fragments from RNA-dependent RNA polymerase, protein S and deletion zone (in blue), and by the human miR hsa miR 451a (in red). Some fragments in red contain deleterious mutations of protein S: N501Y, E484K and D614G (base mutated in green) and in green come from 5′- and 3′-UTR parts of SARS-CoV-2 genome. Probability of hybridization by chance of length 8 (resp. 10) in a sequence of 624 nucleotides equals 0.04 (resp. 0.005), hybridizations T-G and G-T counting for ½.

An example of the existence of accumulation of small RNA fragments exists in Sclerotinia sclerotiorum infected with the SsHV2-L virus, and these small RNAs derived from the virus measure approximately 22 nt, the same length as the miRs, suggesting a cleavage by a protein Dicer-like [51]. Regarding SARS-CoV-2, such an influence on protein translation has already been described [53,54,55,56,57,58,59,60,61,62,63,64,65], causing observed effects on the concentration of certain proteins, such as a dramatic decrease in hemoglobin [66,67,68,69]. If we assume that short RNA subsequences (about 20 nucleotides long), from the genes of the SARS-CoV-2 virus, can bind to Argonaut proteins and hybridize the mRNA of key human proteins, involved in metabolisms important as oxygen metabolism, it follows that recombinations, mutations and/or deletions observed in the SARS-CoV-2 genome (such as those which appeared in the United Kingdom, South Africa, France, etc., or spontaneously in vitro [70,71,72,73,74,75,76,77]) can reinforce the possible existence of RNA fragments, possibly capable of hybridizing for example the mRNA of subunits of hemoglobin, the gamma-globin (Figure 6) and the beta-globin (Figure 7), impacting the oxygen transport in infected patients.

### 4.2. Hybridizing Power of SARS-CoV-2 Viral RNA Fragments

**Figure 8:**
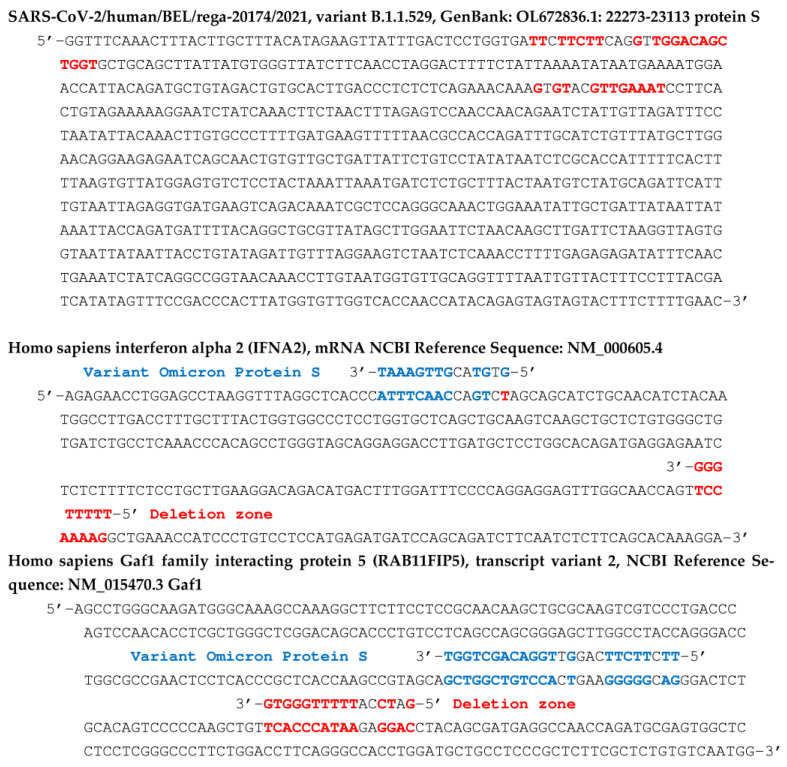
Top: partial sequence of the gene of S protein of the Omicron variant of the SARS-CoV-2 virus with indication (in red) of the subsequences hybridizing the human interferon gene and the anti-aging human Gaf1 gene. In the middle (respectively, bottom), the corresponding hybridization of human interferon gene (respectively, anti-aging human Gaf1 gene) by parts of the protein S gene from its deletion zone (in red) and from its variant Omicron (in blue).

In the following, we focus on viral RNA sequences of the miR type, playing the role of possible germ for mRNA hybridization of proteins participating in fundamental physiological mechanisms, such as the transport of oxygen by the hemoglobin or the induction of immune function protein synthesis. The hybridization seed, if it comprises at least 8 consecutive nucleotides complementary to those of a target mRNA, can cause the elongation of the corresponding protein to be stopped in the ribosome, therefore the inhibition of its biosynthesis [78,79,80,81,82,83,84,85,86] or as tRNA fragments, viral RNA fragments can compete with mRNA for ribosome binding [87]. In the search for hybridization seeds, we have selected RNA sequences from different databases [88,89,90] using the classic BLAST software. For example, we have already noticed in a previous work [62] that miR 129-5p was a known inhibitor of the biosynthesis of gamma-globin 2, a subunit of human fetal hemoglobin, replaced in adults by beta-globin, also regulated, like the other subunit, alpha-globin, by several miRs, including miR 451a [91,92,93,94,95,96,97,98,99,100,101,102,103,104,105,106,107,108,109,110]. The search for hybridization seeds having possibly the same inhibition potential as miRs 129 -5p and 451a thus led to the identification of two subsequences of ORF10 and S protein genes from SARS-CoV-2 genome. Figure 6 shows the subsequences of the mRNA of S and ORF 10 proteins thus identified as possible biosynthesis inhibitors of gamma-globin 2. Figure 8 shows, for its part, two hybridizations by subsequences of the gene of the S protein of Omicron variant of SARS-CoV-2, parts of human interferon and anti-aging human Gaf1 protein mRNAs.

Figure 7 shows concordances of other viral RNA fragments from the RNA-dependent RNA polymerase gene of the SARS-CoV-2 genome, with the beta subunit of the human hemoglobin gene. This concordance is also present in certain variants of SARS-CoV-2, such as that comprising the N501Y mutation, qualified as deleterious, because it increases the hybridizing power of a seed sequence in the initial gene (before mutation) of the RNA-dependent RNA polymerase. Inhibition of beta-globin biosynthesis, involved in oxygen uptake by hemoglobin, could cause a decrease in oxygen flow to the tissues.

In Figure 7 and Figure 8, the 5′- and 3′-UTR parts of the viral genome which have been proved to be deleted in some COVID-19 patients [87] from which possibly hybridizing fragments have been extracted are, respectively:

5′-C**AATCTTTAAT**CAGTGTGTAACATTAGGGAGGACTTGAAAGAGCCACCACATTTTCA-3′ and

5′-**GTAGATCTGTTCTCTAAA**CGAACTTTAAAATCTGTGTGGCTGTCACTCGGCTGCATGCTTAGTGCAC

TCACGCAGTATAATTAATAACTAATTACTGTCGTTGACAGGACACGAGTAACTCGTCTATCTTCTGCAGGCTGCTTACGGTTTCGTCCGTGTTGCAGCCGATCATCAGCACATCTAGGTTTTG-3′.

**Figure 9:**
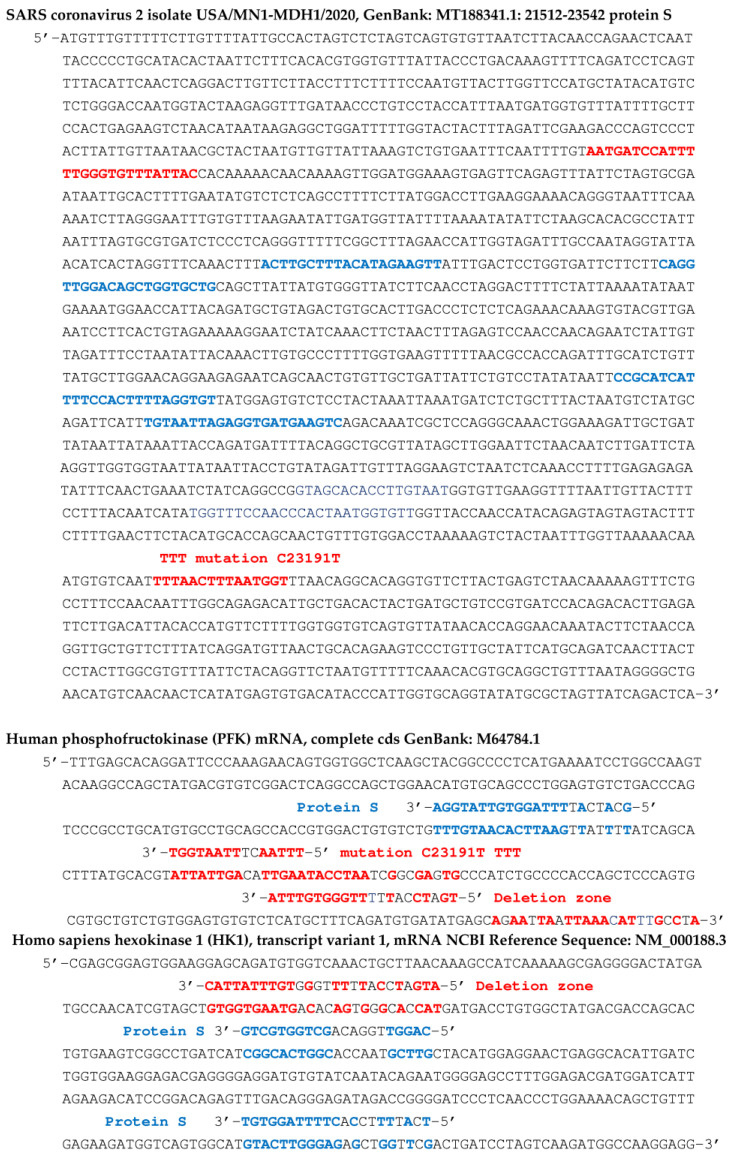
Top: partial sequence of the S protein gene of the SARS-CoV-2 virus. The first blue subsequence of length 20 hybridizes adult hemoglobin subunit beta-globin (HBB)80, phosphofructokinase (PFK), hexokinase 1 (HK1), serpin family C member 1 (SERPINC1) and C-reactive protein (CRP). The second of length 22 hybridizes a mRNA subsequence of the hemoglobin subunit gamma-globin 2 (HBG2). In the middle (respectively, bottom), possible target subsequences from PFK (respectively, HK1) gene. Blue color indicates sequences of Protein S mRNA anti-matching human genes and red color indicates deletion or mutation zones of Protein S mRNA.

In Figure 6, Figure 7, Figure 8 and Figure 9, we have indicated fragments coming from a particular zone of the protein S gene prone to deletions [67]: 5′-AATGATCCATTTTTGGGTGTTTATTA-3′.

Figure 9 shows that the first blue 20 nt subsequence of protein S gene can possibly hybridize mRNAs of adult hemoglobin subunit beta-globin (HBB), phosphofructokinase (PFK), hexokinase 1 (HK1), serpin family C member 1 (SERPINC1) and C-reactive protein (CRP). The second blue subsequence of the S protein gene, of length 22, can possibly hybridize mRNA of hemoglobin subunit known as gamma-globin 2 (HBG2) [91,92,93,94,95].

Starting from proteins linked to the symptomatology of COVID-19, we have previously shown [51] that the existence of RNA fragments of the viral genome could be candidates of the miR type (“miR-like” hypothesis) inhibiting the synthesis of various proteins, such as those associated with olfactory, nicotinic, immune (interferon) receptors, with the beta subunit of adult hemoglobin [91,92,93,94,95] linked to oxygen transport, or eventually with glycolysis or cellular aging mechanisms. These effects can be marginal, but cumulative, and can play a role in the medium term, for example in the long COVID syndrome. Moreover, the English, Brazilian and South African variants, which have numerous mutations, including the deleterious mutations E484K, N501Y and D614G in the SARS-CoV-2 protein S RNA sequences, would have, under the miRNA-like **hypothesis**, a greater inhibitory power than the initial sequences of the “wild” virus, because, when they present an additional hybridizing nucleotide. It is therefore possible that the deleterious effects observed on contagiousness and mortality due to these new variants are caused by the presence of RNA fragments carrying these mutations. For example, the mortality rate of the Delta variant is on average 1.64 times higher than those of the previous variants in the United Kingdom [96] and the basic reproduction number R0 of the Omicron variant is estimated on average 4.2 times higher than that of the Delta variant in South Africa [97].

Other examples could be provided by the phenomenon of thrombosis observed after administration of the AstraZeneca^®^ [98] vaccine, possibly due to the inhibition of the synthesis of anti-thrombotic proteins, such as SERPINC1 and CRP, or by the specific mortality of diabetic patients with COVID-19 [99,100], possibly due to a decrease in the concentration of glycolytic proteins, such as PFK and HK1. SARS CoV-2 would therefore have a potential “cluster bomb” effect and mRNA vaccine therapies could present a comparable risk, if injected mRNA is fragmented in the target cells. The natural response to the overactivity of miRs is their blockage by circular cytoplasmic RNAs or other antagomiRs (or anti-miRs, i.e., oligonucleotides preventing the binding of miRs to mRNA molecules). AntagomiRs are used to inhibit endogenous or exogenous miRs: they act as sponge-like decoys and promote the hybridization of miRs to the complementary (by Crick-Watson type pairing) of their own sequence and not to the sequences of mRNA of proteins involved in vital metabolisms, thus regulating their inhibitory power [101,102,103,104,105,106,107,108,109,110]. This observation could lead to investigations towards RNA therapy (in addition to effective drug therapy [111,112,113]), which could represent an answer to the production of inhibitory fragments, derived from the viral RNA genome and possibly from vaccine RNA, since we cannot rule out the plausible existence of small pathogenic miR-like fragments, resulting from the cleavage action of the viral or vaccinal RNA by the cytoplasmic nucleases present in the host cells.

## 5. Biodistribution of mRNA Vaccine

The biodistribution studies are commonly considered alongside pharmacokinetic studies, since they share some common parameters used to discern the rate and the extent of drug distribution after its application. The key parameters considered by the majority of authors are: peak drug concentration (Cmax); time needed for the drug to reach peak concentration (Tmax); area under the activity curve (AUC); time needed for 50% of the drug to be eliminated (half-life t1/2); and the average time the drug remains in the blood or tissue of interest (mean residence time MRT) (see [114] for a report by EMA including this topic).

Currently, pharmacotherapy uses a panoply of therapeutic agents, ranging from small molecules to more complex cells, including cellular fragments. These molecules/cellular fragments/cells can be natural or chemically or genetically modified. Among these 3 divisions, therapeutic agents can be classified according to whether they are conventional drugs or biological therapeutic agents such as proteins, oligonucleotides and other biomolecules (either derived from cell biology or derived from organic synthesis). Whatever the therapeutic agents chosen, all pose problems of delivery and of biodistribution in the organism from the site of delivery to the site of targeted action. In order to overcome this problem, several drug delivery systems (DDS) have been developed such as liposomes, nanocarriers, drugs conjugated to others having particular affinities with certain tissues, etc. Moreover, in some cases, the DDS themselves have additional functions like therapeutic actions [105]. Each delivery system has its pros and cons. Pharmacokinetics is defined as “what the body does to the drug” and is described using four critical processes: absorption (A), distribution (D), metabolism (M) and elimination (E) (ADME). The interactions between the molecule (therapeutic agents or DDS) and the organism control the relative rates and effectiveness of each of these processes and the bodily compartments involved [115].

**Figure 10:**
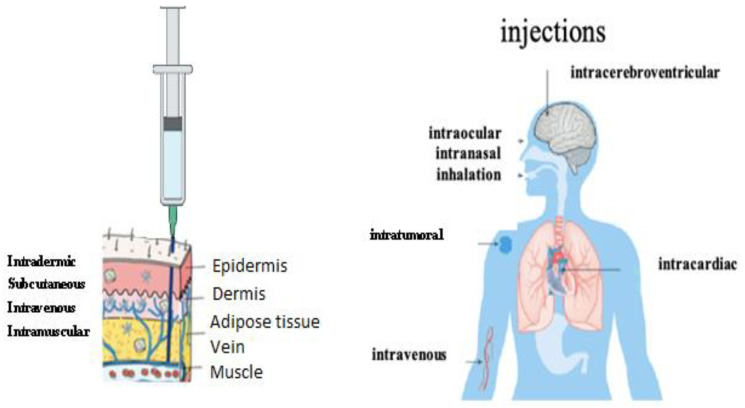
Administration routes of LNP-ARNm [126].

The absorption step is directly linked to the mode of administration chosen, so that the molecule reaches its target (Figure 10). In case of mRNA vaccine, the target is any cell, since the encapsulating liposome is not targeting and not recognized by the immune system. It is easy to understand that the absorption stage can be direct and complete in case of intravenous administration, or longer, or even incomplete (partial bioavailability) in oral administration, since the drug must first cross the intestinal lumen, the portal system, then the liver, before reaching the general circulation. Some molecules cross these compartments easily, other molecules may require specific transporters to pass. Since RNAs have a polyanionic character due to their structure containing PO_4_^3−^ phosphate groups, the cell plasma membrane is difficult for them to cross because of their negative electrochemical potential [116]. This is why the choice was made to encapsulate the mRNA in a liposome, which allows its facilitated entry, by endocytosis, into the cell. Finally, the drug is eliminated from the body either in unchanged form or after biotransformation. This irreversible excretion step is most often done in the urine (renal route) or in the feces (biliary route). Some molecules can be reabsorbed, which defines an enterohepatic cycle. In the case of mRNA vaccination, the elimination of mRNA takes place in the cytosol via nucleases. During vaccination by the intramuscular route, the liposome can, due to its chemical properties, cross the capillary barrier and disseminate itself in all the tissues [117,118].

By this route, the liposome reaches the general circulation, without having to pass through the portal system and the liver. Absorption will be more or less complete and more or less rapid depending on the physicochemical properties of the liposome: injected into the arm, it does not remain locally, if it is not targeting, and diffuses very quickly (between 15 min and 4 h maximum throughout the body) and can, therefore, be incorporated into any cell of any organ. Before the COVID-19 epidemic, only one drug using encapsulated mRNA was approved by the FDA, followed by the European Commission in August 2018, the Patisiran^®^, using LNPs encapsulating siRNAs (anti- senses). This treatment required more than 15 years of development, it ensures a prolonged release of siRNA in the tumor region, thanks to the biodegradable polymer PLGA [119]. We can see that this technology is very recent in humans and is mainly used in oncology, for patients with a vital prognosis. It is significantly different from current mRNA vaccine technology, with a distinct purpose.

## 6. Goal of Vectorization

One of objectives sought in the vectorization in mRNA therapies is to make the oligonucleotides sufficiently stable within the organism and to avoid their rapid degradation in the bloodstream. The improvement of their addressing, thanks to vectorization, avoids their premature elimination.

The ideal vector should meet the following criteria [107]:

- It must be easy to produce on an industrial scale by simple and safe methods. It should also be as inexpensive as possible.
- It must be non-immunogenic and thus allow repeated administration.
- It must be specific for the targeted cells.
- It must be able to adapt to the size of the oligonucleotide to be transported.
- It must be internalized by the cells and allow the release of the oligonucleotide into the cell (transfection phenomenon).
- It must break down and be easily eliminated from the body.

Currently, the ideal vector of mRNAs does not exist, despite numerous researches and advances in the field. A suitable vector needs to be chosen according to the physico-chemical nature of the oligonucleotide to be transported.

### 6.1. Differences between “Classic” Vaccines and New mRNA Vaccines Encapsulated in a Nanoparticle Liposome

There is a major difference between the usual vaccines imagined by Pasteur and the new mRNA and DNA vaccines. In the usual vaccines, we inject either a recombinant protein (technology used by Sanofi^®^ and GSK^®^), or an adenovirus (technology used by Johnson & Johnson^®^ and AstraZeneca^®^), or an inactivated virus (technology used by Valneva^®^ and Sinopharm^®^). Thus, the antigen is directly in contact with the tissues at the injection site and is quickly taken up by the immune system. Because of this rapid management, antigens, even cytotoxic ones, do not have time to spread to all the tissues, which limits the adverse effects related to the toxicity of the surface proteins of the pathogenic agent. Similarly, for new DNA vaccines, the adenovirus quickly recognized by the immune system does not have time to spread to all tissues. However, thrombocytopenia-like thromboses have been consistently reported following administration of adenoviral gene transfer vectors in animal models [120,121,122].

Vaccine-induced thrombotic thrombocytopenia (VITT) has been reported following vaccination with Astra Zeneca^®^ AZD1222 (the estimated rate of thrombocytopenia syndrome within 14 days after the first dose is equal to 8.1 per million of vaccinated people compared to 2.3 per million of vaccinated people at the second dose of AZD1222) and with the Johnson & Johnson^®^ Ad26 [123]. In the case of mRNA vaccines, host own cells make the antigen (here the Spike protein). The synthetic mRNA is encapsulated in a nanoparticle liposome (LNP), which does not have antigenic proteins on its surface, nor molecules targeting particular cells. LNPs, peptides and especially polymers, seem to be at the center of attention, as evidenced by the large and growing number of publications that have cited them for the past fifteen years, in the field of polymer chemistry research. Size, charge and surface of these nano vectors are important parameters to obtain a targeted delivery, with a minimal quantity of vectors [124].

### 6.2. Liposome Physical Properties

The choice of nano-vectors of size between 50 and 250 nm can promote the extension of their circulation in the body, by reducing renal excretion and opsonization. In addition, size plays a role in the accumulation of nano-vectors in certain tissues and their cellular internalization. LNPs, always between 50 and 250 nm in size, passively accumulate in tissues with high vascular permeability [124]. The LNP used in vaccination measures between 60 and 100 nm. A size of less than 100 nm allows diffusion through the pores of the capillaries located at the level of the muscles and allows the penetration of BBB [125]. Due to its size, the LNPs used can thus easily reach the bloodstream.

### 6.3. Liposome Chemistry Properties (LNP)

The synthetic mRNA, encapsulated in liposomes developed by the Pfizer/BioNtech^®^ and Moderna^®^ laboratories, by their physico-chemical nature, diffuses easily into the tissues, without risk of being destroyed by the immune system. LNPs can be injected by different routes [126]: nasal, intradermal, intramuscular, intravenous, etc. (Figure 10).

The Pfizer/BioNtech^®^ Laboratory is based on an LNP developed in 2016, which would have a strong affinity for dendritic cells, without however being targeting and exclusive to these cells [127]. When injected intravenously for immunotherapies in oncology in animals, it has nevertheless been shown that this type of LNP goes preferentially to dendritic cells. Targeting of dendritic cells is still in its infancy. Indeed, in the subcutaneous route, the LNP is more likely to reach its target before being endocytosed by other cells, which is not the case in the intramuscular route. The chemical properties are due to the fact that the typical LNP contains four different parts: (i) the ionizable lipid, which allows it to interact with the cell plasma membrane [128], (ii) the stabilizing agent (cholesterol), (iii) a phospholipid allowing the fusion with the lipid bilayer of cell membranes, and iv) a polyethylene glycol (PEG) [129,130,131], anchoring polymer, which increases the stability in a biological medium and facilitates the manufacture of small, homogeneous LNPs, typically 50–100 nm in diameter, making them less likely to activate the immune system (Pfizer/BioNtech^®^ and Moderna^®^ warn of the allergic risk concerning this PEG). Ionizable (cationic) lipid is essential for intracellular entry of mRNA, as it enhances mRNA encapsulation, increases the extent of interaction between mRNA and cell membrane despite their charge negative, and may even aid in endosomal escape into the cytoplasm, whereby mRNA enters the endosome, so that it can be translated into the immunogenic encoded protein.

Therefore, the liposomes used have nothing targeting, despite a particular affinity for dendritic cells, and their physicochemical nature allows a very wide distribution in the human body. Recently, a preclinical study using the LNP from Acuitas Technology^®^ (this LNP is used by the Pfizer/BioNtech^®^ laboratory in the anti-COVID-19 vaccine) demonstrated, in mice, that it triggered a highly inflammatory response [119]. Once in the bloodstream, liposomes can reach any organ and be endocytosed by their cells. The intensity of the bond between liposome and cells constitutes one of the factors modulating tissue distribution. Finally, the amount fixed can be different from one tissue to another, depending on the affinity. Take the example of brain tissue: it is rich in lipids and has a high affinity for fat-soluble molecules (such as LNPs). The distribution at the level of this organ will therefore be greater for these molecules. The nature of the LNP used could allow the passage of the blood–brain barrier [126], as well as the placental barrier. The conditional marketing authorizations for the Pfizer/BioNtech^®^ and Moderna^®^ vaccines contain a paragraph dedicated to the biodistribution study of the vaccine. These documents are public and accessible on the website of the European Medicines Agency (EMA) [114]. We will detail them below.

### 6.4. LNP Safety Profile

The safety profile of lipid-mRNA nanoparticle formulations correlates with the lipid components and mRNA molecules. Lipid components can activate host immune responses, after systemic or local administration. For example, LNPs with PEGs (as is the case with anti-SARS-CoV-2 vaccines) can induce hypersensitivity reactions, by stimulating the complement system [132,133]. In addition, anti-PEG antibodies could cause rapid systemic clearance of PEGylated nanoparticles subsequently administered, through an accelerated blood clearance phenomenon, which can alter the bioavailability and biodistribution of the drug encapsulated in these nanoparticles and, thus, cause undesirable effects [132,133]. To mitigate safety concerns, many natural and synthetic polymers have been studied as alternatives to PEG, several of which being evaluated in clinical trials [131,132]. Cationic and ionizable lipids have also been reported to stimulate the secretion of pro-inflammatory cytokines and reactive oxygen species [134,135]. Although immunogenicity of these lipids is not yet fully understood, system of complement and Toll-Like receptors may participate in innate immune activation. Cytotoxicity of lipid materials also poses safety concerns depending on dose, lipid properties and cell types [135,136]. In vivo application of lipid nanoparticles has been reported to induce liver and lung damage in rodents, which may relate to cytotoxicity of the materials and induction of pro-inflammatory factors. To improve the biocompatibility of LNPs, biodegradable lipids can be used.

### 6.5. Conditional Marketing Authorization for the Pfizer/BioNtech^®^ Vaccine [137]

Synthetic mRNAs encapsulated in LNPs can reach many organs, such as the spleen, heart, kidneys, lungs and brain. The mRNAs were found in the ovaries and the testicles in small quantities, during the biodistribution studies of this vaccine after 9 days, which implies that endocytosis is not as rapid as supposed. In rats, it has been shown, by integrating very high concentration radio-labelled mRNAs into liposomes (to observe the contrast), that LNPs are found in all organs and not only in antigen/antibody meeting places, such as the spleen, and are not exclusively endocytosed by dendritic cells. The half-life of LNP in the body is 20-30 days and complete elimination is approximately 4 months.

### 6.6. Conditional Marketing Authorization for the Moderna^®^ Vaccine [138]

Vaccine mRNAs are detectable in a wide variety of organs: brain, heart, lungs, eyes, gonads. It is impossible to know their bioavailability in the ovaries, as no female rats were included in the Moderna^®^ vaccine biodistribution study. Even though the concentration in humans is lower than that observed in rats, we cannot conclude with certainty that only muscle or dendritic cells endocyte the liposomes of Moderna^®^ vaccine.

## 7. Discussion

### 7.1. How to Overcome Some Obstacles in the Development of mRNA Vaccines for Diminishing the Adverse Effects

**Figure 11:**
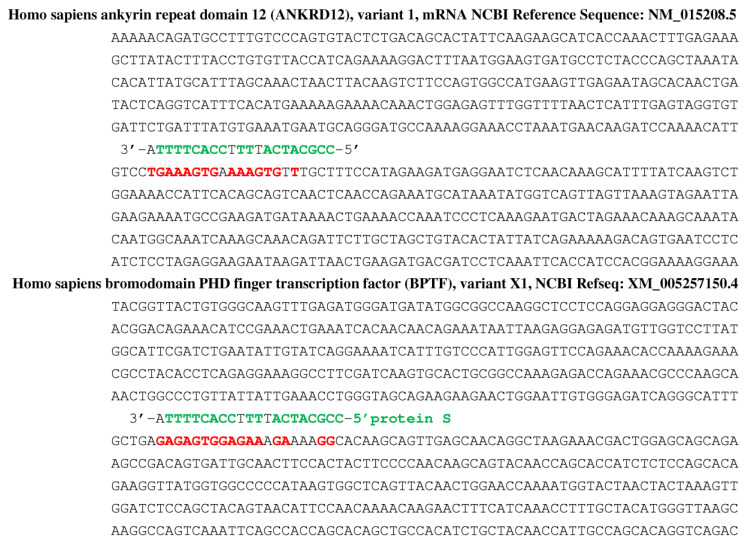
Hybridization of fragment (in green) from the protein S (just those having one putative miRNA-like inhibitory effect on beta-globin in Figure 7) on two circular RNA, hsa_circ_0107544 and hsa_circ_0001195, coming from human ankyrin- (top) and bromo-domains (bottom) [150]. Red color corresponds to the human gene sequences anti-matching a protein S sequence.

Extracellular vesicles (EVs) constitute an intercellular communication network. Small vesicles are formed inside the endosome, with same size as a virus, and exocytosed into the extracellular space [139,140]. EVs can deliver active biomolecules, such as small RNAs (coming from mRNA, tRNA and rRNA molecules), peptides and lipids [141,142,143,144,145]. A virus infects cells which in reaction secrete a large amount of EVs, which contribute to the orchestration of the cellular response to the infection [142]. Numerous recent papers [143,144,145] emphasize the role of host miRs in this defense, in particular in the cross-immunity response [146,147] and they propose to use dedicated EVs containing for example miRs inhibiting the viral proteins biosynthesis as anti-viral therapy [148,149]. In the present paper, we have more focused on the existence of virus or vaccine RNA fragments, which can be present in EVs or in host cells cytoplasm (Figure 11).

**Figure 12:**
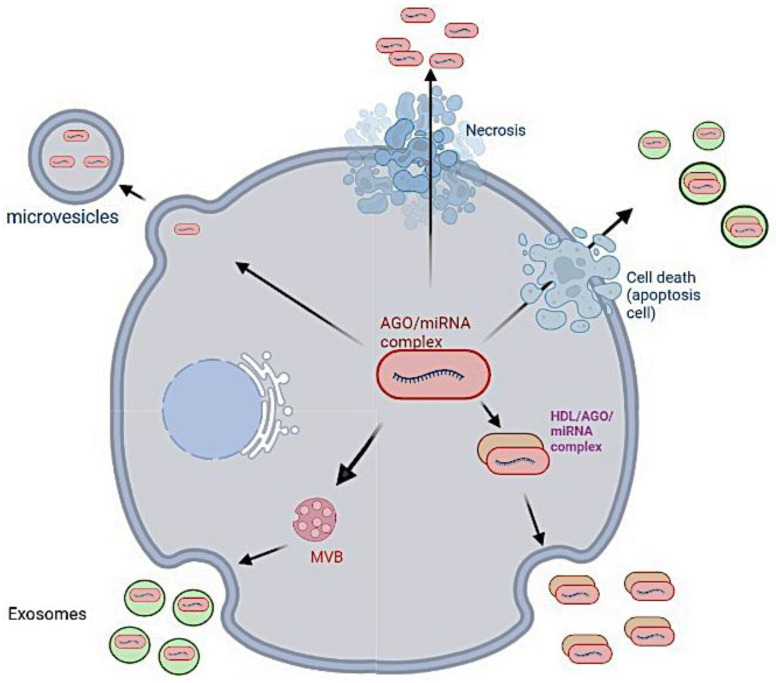
Excretion of the microRNas out of the cell [154].

Fragments of viral genome or vaccine sequence are exported out of the cell (Figure 12) and are found both in wastewaters [151,152] and in impurities formed throughlipid:mRNA reactions [153].

For diminishing the adverse effects of both SARS-CoV-2 variants and vaccines, a new tentative therapy has been proposed. If RNA fragments from viral genome are involved in the pathogenic consequences of the COVID-19, circular RNAs discovered some years ago in virions [155], then as microRNA sponges in a large variety of cells [156,157,158,159]. Circular RNAs [157,158,159,160] have been proposed recently with microRNAs [161,162,163,164,165,166,167,168,169,170,171] as possible actors of antiviral therapy especially in the case of the SARS-CoV-2 if there are fragments in the target cells of the virus originating from deletions and breaks due to the activity of cellular RNases. Figure 11 gives an example of such a fragment from the protein S (just those having one putative miRNA-like inhibitory effect on beta-globin in Figure 7) can be bound to the two circRNAs [150], hsa_circ_0107544 and hsa_circ_0001195, identified in patients with COVID-19 [154] as coming from human ankyrin (top) and bromo domains (bottom) [154].

### 7.2. Optimizing the Development of Vaccines

The development of vaccines often requires years of research and trials to ensure their effectiveness and safety. In the case of COVID-19, the two mRNA vaccines took less than a year to develop and deploy. Extraction of neutralizing antibodies from COVID-19 patient serum verified the strong immunogenicity of complete protein S, the receptor binding domain (RBD) and N-terminal domains (NTD) being also immunogenic. Therefore, immunizing against the complete S protein, rather than just one of its structural units, may lead to a better response, which is unaffected by genetic drift [66]. The other side of the coin is the possibility of inducing a facilitative antibody response (ADE), which may aid in variant selection [160]. Structurally modified COVID-19 mRNA vaccines show good tolerance and may be better at eliciting a rapid antibody response. They provide development flexibility, as any protein can be made from mRNA, without changing the production or application procedure. Optimization of structural features of mRNA, in particular the 5′ cap, 5′ and 3′ UTR regions, coding region and Poly(A) tail increases mRNA control over immune responses, which results in higher translation efficiency. The length of the Poly(A) tail means that mRNA can be translated multiple times. Consequently, it is impossible to know what is the dose of Spike proteins synthesized after each vaccination, no more than its pharmacokinetics and biodistribution over time, whereas, for classic vaccines, we know the exact dose of recombinant antigens or of attenuated virus injected, as well as their dissemination and elimination. The changes made are not without consequences on the risk of potentially serious adverse effects. The bioavailability of LNP is not controlled and there is no guarantee that LNP is endocytosed only by muscle or dendritic cells at the injection site. The fact that LNP can be endocytosed by cells of critical organs (brain, heart, ovaries), which do not regenerate or very little, is highly problematic. Indeed, the cells of these organs, which express the Spike protein on their surface, are then destroyed by phagocytosis via macrophages, a response that results from inflammation linked to innate immunity. The support by the immune system not being immediate, and before the Spike protein will be finally exocytosed in the extracellular environment and joins the blood system, it can then bind to the ACE2 and NRP1 receptors of many cells and thus induce the same problems than those seen in severe infection. It would be wise to develop vaccines using LNPs that specifically target antigen-presenting cells (APC). There are several possible targeting delivery strategies such as adding an antibody to the surface of the LNP that would be specific for a target cell or increasing the affinity of the LNPs with the cellular microenvironment at the site of injection, by acting on the redox potential, the enzymatic activity or even the pH [161,162].

Due to the modifications introduced into the nucleotide sequence coding for the Spike protein resulting from this technology, this protein can adopt several spatial conformations. This property implies a variable and sometimes underperforming and/or altered immune response. Several categories of antibodies can then result, neutralizing antibodies, as facilitating antibodies.

During the development of mRNA vaccines, cross-immunity was completely overlooked. There are anti-coronavirus antibodies and many epitopes common to different endemic coronaviruses, which are conserved in the SARS-CoV-2 genome. Vaccination does not take into account pre-existing cross-immunity [160,163,164] and current protocols neglect acquired immunity following natural infection. They are based on the declaration of an identified infection (with a PCR or symptomatic test) and not on the exact level of antibodies. This should be an option to follow, which remains to be defined until there are dedicated clinical studies to establish the correlates of protection in vivo from ex vivo measures such as the protective threshold rate (by measuring the neutralizing capacity or any other biological variable), with respect to the infection leading to the pathology.

Young individuals are those whose cross-immunity is still active, causing a strong response to vaccination and the appearance of adverse inflammatory effects, particularly at the cardiac level. It would be interesting, in this case, to look both into the viral or vaccine RNA fragments and to small host RNAs, for knowing their short-term effects on metabolism, creation of variants and possible preventive or therapeutic efficacy [153,165,166,167,168,169,170,171,172,173,174,175,176,177].

In order to overcome the problem of vaccine efficacy from one variant to another, It will be interesting to develop a polyepitope-based vaccine, which would contain the nucleocapsid antigen (N) which mutates very little from one variant to another, in order to elicit polyfunctional T cell responses and cross-reactivity.

On the other hand, as previously discussed, growing concerns about PEG hypersensitivity may limit the usefulness of pegylated lipids for therapies requiring chronic administration. Indeed, PEGylated constituents are also immunogenic, inducing anti-PEG IgM and IgG, which are responsible for the accelerated blood clearance and induction of complement activation-related pseudo-allergies. Inflammatory complications were observed with localized delivery of small mRNA–LNP dosages during COVID-19 vaccination campaigns and would be amplified with higher, chronic dosing [178]. Current research is focused on further optimization of pegylated lipids or on the development of different stealth lipids, such as polysarcosine-conjugated lipids which show great promise [179]. To reduce the allergic effects, in-depth studies could be carried out on the population to be vaccinated in order to personalize the recall and booster policies [180,181] by targeting sub-populations at risk, specified by identifying their risk factors (allergy, age, history families, comorbidities, geo-climatic and socio-economic environment [182,183,184,185,186,187,188,189,190,191,192,193,194]).

## 8. Conclusions

A wide distribution of vaccine mRNA in organs can lead to systemic adverse effects and, therefore, there would be a need in the future to develop lipid nanoparticles allowing targeted delivery of mRNA vaccines in specific cells and to deepen the studies on their biodistribution, their bioavailability and their toxicity in animal and human models, in order to refine the doses by calculating their own risk-benefit balance. Hence, we recommend (i) adapting the mRNA of vaccines to the least mutated virus proteins and (ii) personalizing its administration to these categories of chronic patients at risk most likely to suffer from adverse effects linked to the inhibition of metabolisms affected by their pathology. A possible stratification of these categories could be obtained by crossing 3 age groups (0–20, 20–60, ≥60) with 3 chronic pathologies: obesity-diabetes, respiratory failure and cardiovascular diseases.

## Figures and Tables

**Table 1:** Example of amino-acid modification on the mRNA sequence of Pfizer/BioNtech^®^. The red bold color indicates the modified amino-acids.

Aminoacid	L	D	K	V	E	A	E	V	Q	I	D	R	L
Virus	CUU	GAC	AAA	GUU	GAG	GCU	GAA	GUG	CAA	AUU	GAU	AGG	UUG
Vaccine	CΨG	GAC	CCΨ	CCΨ	GAG	GCC	GAG	GΨG	CAG	AΨC	GAC	AGA	CΨG
Aminoacid	L	D	**P**	**P**	E	A	E	V	Q	I	D	R	L

## Data Availability

All the data are coming from public databases: Nextstrain. Available online: https://nextstrain.org/ncov/Europe (accessed on 10 November 2022); Nuccore. Available online: https://www.ncbi.nlm.nih.gov/nuccore (accessed on 10 November 2022); Genecards. Available online: https://www.genecards.org/cgi-bin/carddisppl?gene=HBB (accessed on 10 November 2022); Mirbase. Available online: https://www.mirbase.org/cgi-bin/mirna_entrypl?acc=MI0000093 (accessed on 10 November 2022); EMA. Available online: https:/www.ema.europa.eu/en/documents/assessment-report/NCBI (accessed on 10 November 2022); NCBI. Available online: https://www.ncbi.nlm.nih.gov/nucleotide (accessed on 10 November 2022); ACS. https://www.acs.org/content/acs/en.html (accessed on 10 November 2022).
